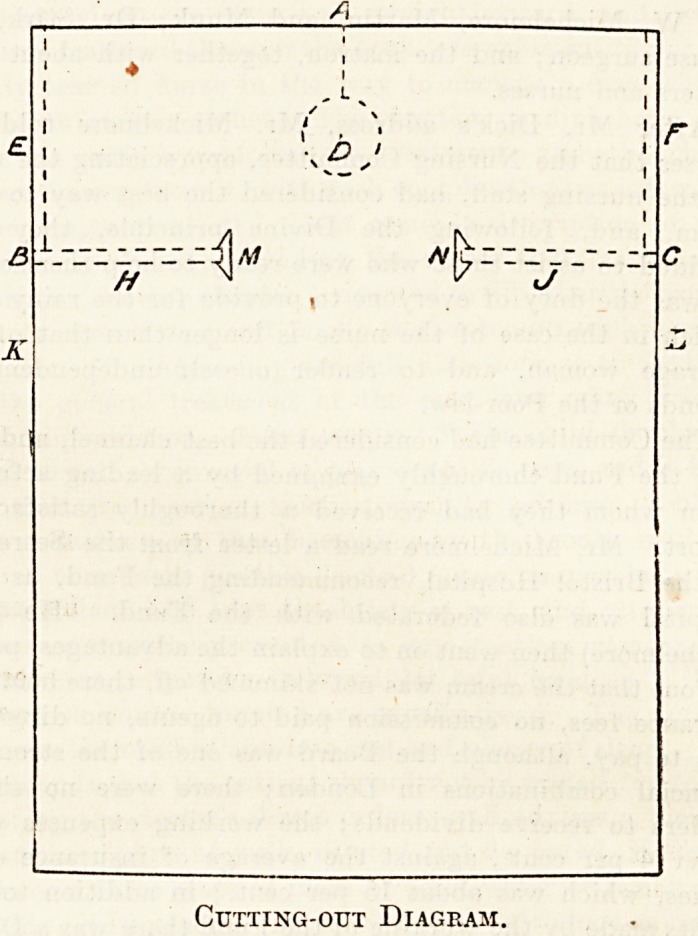# The Hospital. Nursing Section

**Published:** 1906-03-17

**Authors:** 


### The Hospital.

**Nursing Section.**

Contributions for "THE HOSPITAL," should be addressed to the EDITOR, "THE HOSPITAL" NURSING SECTION, 28 & 29 Southampton Street, Strand, London, W.C.

No. 1,016.—VOL. XXXIX. SATURDAY, MARCH 17, 1906.

### Notes on News from the Nursing World.

### RETIREMENT OF MISS SIDNEY BROWNE, R.R.C.

WE understand that Miss Sidney Browne will leave the Army on April 4. She retires from the post of Matron-in-Chief of Queen Alexandra's Imperial Military Nursing Service only because of the age limit, which, whatever convenient purposes it may serve, seems quite absurd as applied to a lady who is in the prime of life, with her vigour and capacity for administration absolutely unimpaired. The period of Miss Browne's association with the service will always be remembered with satisfaction, and the last new rule which has come into operation under her auspices merits recognition. The promotion of sisters to the rank of matron cannot now take place until they have passed a special examination, which includes the answering of written and oral questions, and the delivery of a lecture of forty-five minutes suitable for the instruction of orderlies. This examination may take place five years after they become sisters. The Board of Examiners is to consist of a principal matron and two military matrons, except for stations abroad, when a board under arrangements made by the Director-General is to be appointed to conduct the oral portion of the examination, the written portion being supervised by a local board consisting of a matron and two sisters. To enable sisters to procure the necessary certificate of administrative capacity, those of five years' service and over may, at their own request, as far as circumstances admit, undergo a two months' course of special instruction in the duties of a matron. For the rest, every sister who offers herself for examination will be required to show that, either by means of special instruction or through having acted as temporary matron for two months, she is thoroughly cognisant of the obligations she will be required to discharge as matron; and though we assume that this has practically been the case hitherto, under the new order there can be no question of promotion by favouritism. Sisters who know their work will not find it difficult to pass the examination, and those to whom it offers impediments cannot be fitted to fill the position of matron.

### THE IMPERIAL MILITARY NURSING SERVICE.

WE are officially informed of the following changes in Queen Alexandra's Imperial Military Nursing Service: Miss A. Y. St. Clair, Miss C. A. Coats, and Miss D. M. Smith have received appointments as staff nurses. Miss E. G. Barrett has been posted to the Royal Infirmary, Dublin, and Miss A. S. Siddons to the Military Hospital, Gibraltar—both staff nurses. The appointments of Miss M. Antrobus, Miss M. C. E. Newman, Miss M. Barton, Miss E. C. Ellis, and Miss F. M. Tosh, as staff nurses, have been confirmed. Miss P. Mactairish has been appointed a nursing sister in Queen Alexandra's Military Nursing Service for India.

### THE FIRST MATRON OF KING EDWARD VII. SANATORIUM.

THE first matron of King Edward VII. Sanatorium at Midhurst has been selected in the person of Miss Blanche Trew, a distinguished nurse who possesses the Order of the Royal Red Cross in recognition of her services during the war in South Africa. Miss Trew, in addition to her South African experience, has been connected with several important institutions, from University College Hospital at which she was trained, to the Royal Cornwall Infirmary at Truro, of which she was matron. She was also for some time assistant superintendent of the Nurses' Co-operation.

### RESIGNATION OF A LONDON MATRON.

AT the Annual Court of Governors of the Royal National Orthopædic Hospital, the Committee received with deep regret the resignation of Miss Frances Hole, the matron, who has been absent on leave through serious illness for more than a year. It had been the hope and expectation of the Committee that prolonged rest and skilled medical treatment would have enabled her to regain her health and resume her duties. Miss Hole, who was matron of the late National Orthopædic Hospital for seventeen years, has come to the conclusion that her state of health will not allow her to undertake the increased responsibilities brought about by the amalgamation, and has therefore resigned her post.

### A PAUPER ATTENDANT AS ASSISTANT NURSE.

A LAMENTABLE instance of carelessness is reported from Rochdale Workhouse Infirmary. At an inquest on Monday, on an inmate who died in the institution, it was stated that while the nurse rubbed the back of the patient, who suffered from heart-disease and kidney complaint, with methylated spirit, to prevent bed-sores, an inmate assisting her struck a match to light his pipe, and a portion of the brimstone fell amongst the bedclothes and ignited the spirit. The patient, who was eighty years of age, died shortly afterwards. But what business had the pauper attendant with a pipe in the infirmary ward? And how was it that the nurse permitted him to strike a match under conditions which were obviously dangerous? Possibly, of course, the nurse had no idea that her assistant contemplated lighting up, and therefore had no chance of using her authority to forbid it, but however that may be, the moral of the accident is the same. If a probationer had been employed instead of a pauper attendant, a disaster so regrettable in the interests of the Rochdale Workhouse Infirmary would not have occurred.

### PROFESSOR OSLER AND TUBERCULOSIS CASES.

The annual meeting of subscribers to the Sarah Acland Memorial District Nurses' Home and the Acland Nursing Home at Oxford was marked by an extremely interesting speech from Professor Osler, who seconded the adoption of the report. In the course of his speech he said that he had seen the work of the Nurses' Home, and could bear testimony to its singular efficiency; and with regard to the district nurses, it astonished him to learn the remarkable number of visits paid by the nurses in the course of a year. When he thought of what the visit of a good nurse meant in a poor house, he doubted whether there was any work done in the city of Oxford which equalled in benefit the work done by these nurses in visiting the sick in their midst. Professor Osler went on to say that he would be glad to see the work grow particularly in one direction. He would like the whole subject of tuberculosis to be dealt with by one special nurse, and he suggested that the citizens should subscribe additionally for an able nurse who would look after the tuberculosis cases in the city, and perhaps in the adjacent district. The proposal is an admirable one, and, coming from Professor Osler, it will, no doubt, command the attention it deserves.

### BELFAST NURSES AND THE PENSION FUND.

At the annual meeting this month of the Society for Providing Nurses for the Sick Poor of Belfast, it was stated that during the year £41 12s. was paid in pensions to nurses, the income of the superannuation fund investments being only £21 2s. 11d. From the report, however, it appears that these pensioners are the first nurses of the Society, who are not connected with the Royal National Pension Fund, to which all the younger nurses are required to belong. It is added that "some years hence, when all the pensions are paid by the Royal National Pension Fund, the Society's general fund will be repaid for its present pension expenditure by the superannuation fund." No difficulty is found in inducing the nurses to belong to the Pension Fund, and the substantial advantages are generally recognised. A fair idea of the useful work done by the Society last year may be judged by the fact that no fewer than 202 cases of consumption and 65 of cancer were nursed.

### A GREAT WORK IN EAST LONDON.

At the annual meeting of the East London Nursing Society, on Tuesday, which, as usual, took place at the Mansion House, Alderman Sheriff Smallman in the chair, its claims were pleaded by more or less representative speakers. On this point our readers do not need to be convinced. It was only a few weeks since that an article describing the work carried on under the auspices of the Society, by a former member of the staff appeared in our columns, which must have satisfied any who may have been in doubt that it merits the warm support of all who are anxious that the sick and suffering poor in the East End should be tended in their own homes by capable and kindly nurses. We are glad to learn from the annual report that the accounts for 1905 show a balance on the right side. But, as it is pointed out, this must be attributed to the sum realised by the entertainment at Grosvenor House last March, and to a legacy of £100. New subscribers are, in fact, urgently needed in order to ensure the continuance of the work on its present lines, let alone its extension, which is greatly desired. Most of the staff are Queen's Nurses.

### A DEFICIT AT BOLTON.

There was one unwelcome announcement in the report of the Bolton District Nursing Association which was adopted at the annual meeting last week. The Association has existed for seventeen years, and for the first time in its history the committee could not make two ends meet in 1905. The deficit was only £32, and as there was a credit balance at the end of 1904 of £26 16s. 10d., the debit balance now stands at but a little over £5. Still, the record is unsatisfactory, and the more so as, except for the profits of a dance which brought in £70, there would have been a considerable difference on the wrong side. Yet the work of the nurses, as set forth by the superintendent, Miss Walker, was all that could be desired. The number of visits paid was 35,995, and the number of cases nursed 1,319. Several speakers testified in warm terms to the value of the charity, and the Mayor begged for increased support from all classes. Unless this is forthcoming a reduction in the staff of nurses was foreshadowed.

### NIGHT NURSES AT WHITECHAPEL INFIRMARY.

The letter of "A Whitechapel Nurse," inserted in our issue of last week, complaining of the sleeping accommodation of the night nurses at Whitechapel Infirmary, of their off-duty time, of the manner in which the meals are served, and of alleged shortness of food, has provoked emphatic and indignant contradictions, which we publish this week with pleasure. The communication of a sister completely traverses all the statements made. There is no doubt as to the *bona fides* of our correspondent, but she now writes to express her regret that her letter was not clear to us, and that her references were intended to be to the sick wards of a workhouse in which she was formerly employed, and not to Whitechapel Infirmary. This frank statement will be as satisfactory to the authorities and staff at Whitechapel Infirmary as it is to ourselves, though we, of course, are sorry that our correspondent's communication should, even for a moment, have reflected unjustly on an apparently admirably well-ordered and well-conducted institution.

### MIDWIFERY IN WORCESTERSHIRE.

The principal feature in the report discussed at the annual meeting of the Worcester City and

County Institution for Trained Nurses was the effect of the addition of midwifery training on the work. This new departure was undertaken at the request of the County Council, who made a grant for each pupil trained. An experienced obstetric nurse has been engaged, and under her the pupils attend cases among the poor in Worcester. Lectures are also given to them by a medical man, and it is hoped that a band of skilled midwives will gradually be sent out to work throughout the county. Sir Harry Vernon, in moving the adoption of the report, commented upon the satisfactory position of the midwifery branch, and said that it was fortunate that their lady superintendent was able to undertake instruction; and Miss Mickie herself stated that the midwives attached to the institution have already attended 80 cases, most of them being amongst the very poor. As superintendent she has the strongest belief in the importance of this work, by means of which it is hoped that many people will be taught to bring up their children in a more sensible and healthy way. She is convinced, from personal experience, that a great deal of the physical deterioration, which is so much talked about, is due to improper feeding in infancy.

### OLD-TIME METHODS.

At the annual meeting of the Cambridge District Nursing Association, Professor Howard Marsh, who moved the adoption of the report, told two anecdotes illustrating old-time methods of nursing. Early in the last century, he said, an old nurse, whose duty it was to take care of patients at night, was in the habit of looking about for a fairly convalescent patient, putting her on duty, and thus ensuring for herself a good night's rest. On another occasion Mr. Abernethy was standing gossiping in the square of St. Bartholomew's; he saw a nurse approaching, and observed to his companion, "See what I am going to do." Lifting his stick he struck the woman on a protuberance in the region of the thigh, remarking, "Ma'am, if that ain't a bottle of gin, I beg your pardon." But it was! Whatever may be urged in deprecation of the modern nurse, it will be admitted that she neither expects her patient to take her night duty, nor carries a bottle of gin about with her.

### THE PRINCESS CHRISTIAN CHILDREN'S NURSES

An appeal for financial support to the Princess Christian College in Manchester has been forwarded to us with a request for notice. The college was founded in 1901 to meet the increasing demand for ladies as children's nurses and to provide the necessary training for educated women who have to earn their own living and who have a natural sympathy with young children. It is stated that 1,800 applications for these nurses were made last year, which attests the reality of the demand, and the money now wanted is said to be needed to discharge a debt incurred through the rapid growth of the College, for further extensions, and for a moderate endowment for the nurseries which is required to supplement the fees of the students. But we think that this admirable institution ought, by this time, like others of its kind, to be self-supporting.

### LADY MARGARET FRUITARIAN HOSPITAL.

On Saturday afternoon the Marchioness of Downshire had consented to hold a reception at the Lady Margaret Hospital, Bromley, but being unavoidably prevented, the matron took her place. The annual meeting followed, when the Vice-Presidents were elected for the coming year. The proceedings took place in the women's ward. Sister Oriel was the "M.C." of the occasion, and the nurses, who are trained in domestic work and fruitarian cookery, as well as in nursing, in their pretty blue striped dresses formed an attractive bodyguard. The speakers included the Honorary Matron, Miss Sharpe, and Dr. Oldfield, who laid stress on the fact that the institution is a "fruitarian" as well as a vegetarian hospital, and he invited the visitors to question any or all of the patients as to their diet, etc., whilst they were in the hospital.

### A PLACE FOR A REST CURE.

Kidbrook House, 78 Shooters Hill Road, Blackheath, the new nursing home for medical, surgical, and maternity cases, was open to inspection last week. The Home, which, as already stated, is under the care of Miss Maud Marks and Miss E. L. Underhill, is a large and commodious residence, and stands well back from the road, and has a nice piece of ground at the rear. Formerly a school, and afterwards inhabited by an Army coach, it has required practically no structural alterations to fit it for its present purpose. There are 12 rooms for patients, with 14 beds. The chief feature is the unusual allowance of windows, most of the rooms, even the smaller ones, having two. These, of course, give a very bright, airy look to the apartments. The operating theatre is fitted up with all the latest appliances, including a Schimmelbusch steriliser. The lying-in ward, which is entered through a smaller room, is quite secluded, and also possesses the advantage of accommodation for a night nurse in case of necessity. The dining-room is very spacious, with three large windows; the kitchens and offices are also of a good size, and convenient. There is a large room in the basement, which it is hoped may later on become a drying and ironing room, there being another building available for a laundry. A cheerful room, to be used for the staff, possesses three windows. Altogether, what with the sunlight flooding the house through its many windows, the artistic colouring, and pretty furniture, the remark of one visitor, "Just the place I should like to come to for a rest cure," quite reflected the general impression.

### NURSES WANTED FOR CHINA.

It is very satisfactory to know that a Chinese woman has come to London to learn nursing in one of our great training schools. Meanwhile, we understand that English nurses are wanted at the Mission Hospital of the Church Missionary Society at Ningpo. Of course they need, in addition to training, a love for the special work which is distinctly arduous. There are a great many major operations at the hospital, and at present the preparations for them, as well as the operation itself, have to be done by the operator.

### The Nursing Outlook.

"From magnanimity, all fears above;
From nobler recompense, above applause,
Which owes to man's short outlook all its charm."

### THE WORK OF THE TRAINING SCHOOLS.

Astonishment has often been expressed that the great training schools throughout the country and especially in London have not formed an association of their own, so as to secure active co-operation and an interchange of views and experience, to enable the school authorities to take an effective part in the organisation of nursing throughout the country. We content ourselves to-day with the statement of the fact, without considering the underlying causes which have produced this inaction. The absence of co-operation is regrettable for the reason that the nurse-training schools under present conditions have failed, and must continue to fail, to exercise that predominant influence in nursing affairs, which their work entitles them to wield. The progress in nursing in recent years, and the improvement in the quality of the education and training now made available for all nurses at the greater schools, at any rate, must ultimately bring about a change of opinion, and thus lead to a closer connection between one school and another.

It must not be forgotten that the matron or lady superintendent in charge of a great nurse-training school has, in fact, to face a multiplicity of duties, of so varied and onerous a character, as to be beyond the strength of any but the strongest and ablest administrators who have qualified as nurses. The infinity of detail in connection with the ward and other work of a great hospital, including the supervision of the housekeeping, and the general working in the various departments or sections, much of the work of which must occupy the thought of the matron, entail an output of energy which very few women are capable of continuously supplying. Every year the demand for new departments and sub-divisions of work, together with the introduction of new subjects or modifications in the curriculum of the training school, require thought and ability on the part of the lady superintendent. The control of a great household and the supervision of hundreds of nurses, with all that it entails, would be enough in itself to exhaust the energies of not a few women of capacity. It follows that where the matron has succeeded in maintaining a high state of efficiency throughout the whole of the departments under her charge in a great hospital, she has given evidence of the possession of qualities of a high order which are as valuable as they are rarely met with.

The office work alone in the matron's department of a great hospital, and the correspondence which it involves, is sometimes even greater than that of the secretary himself. Through this correspondence the matron is brought into contact with workers up and down the country, and has to supply suggestions in answer to questions and to render considerable service to the cause of sound nursing, which has in fact no relation to her proper duties in the institution she may serve. When people grow impatient and accuse the officials of large hospitals of being too self-absorbed and without public spirit, they are apt to forget what a large amount of work many of these matrons voluntarily undertake in educating those who have to carry on different branches of the work elsewhere. A great training school like the London Hospital, as the result of many years' work, necessarily has representatives of its training school holding important appointments in many other hospitals and kindred institutions throughout the country and even abroad. Every such official properly looks to the matron of her training school for assistance in her difficulties, and expects her to help them because of their relatively small experience to deal with conditions which are new to themselves. In this way the training schools render very real service to the institutions and the country, and so to the cause of improved nursing and better conditions for the care and comfort of hospital patients. If it were possible to bring the critics of the nurse-training schools face to face with an experienced worker like Miss Lückes, and to elicit by question and answer how ignorant in fact they are of the infinity of detail which such a matron has to grapple with, and of the continuous assistance which she is rendering to workers in other hospitals, every week throughout the year, they would stand revealed as incapable of exercising the office of critic, which is more often than not self-imposed, and without justification so far as knowledge and experience are concerned. It is a very real gain for any woman who aspires to take high office in the nursing world to have had an opportunity of working under an experineced matron in one of the larger hospitals where the system, when tested by experience, has proved itself to be of the best. The very fact of having had the advantage of being a part of a large organisation, where method is simply a necessity, gives confidence to the worker when placed in a position of responsibility where she has to direct and inspire others. No doubt in this country we want a few of the heads of the great training schools to take an active and personal part in the public business of nursing. But no one whose opinion is worthy of weight will fail to be grateful to the training schools and their officials for the habitual, systematic help now given in a practical way whenever it is asked for by those who have any claim to demand it.

### Abdominal Surgery.

By Harold Burrows, M.B., F.R.C.S., Assistant Surgeon to the Seamen's Hospital, Greenwich, and to the Bolingbroke Hospital, Wandsworth Common.

### AFFECTIONS OF THE STOMACH.

### Anatomy.

The stomach is a hollow muscular organ whose functions are to receive food that is swallowed, and to commence the process of digestion, which will be completed lower down in the intestine. In health, the stomach lies in the epigastric and left hypochondriac regions, but in some abnormal conditions the organ may be displaced downwards, or may be so much dilated as to encroach even upon the hypogastrium. The œsophagus opens into the upper end of the stomach, the opening being known as the cardia, or the cardiac orifice. At the other end the stomach is continuous with the duodenum. The opening between the stomach and the duodenum is the pylorus, or pyloric orifice. The cardiac and pyloric orifices are surrounded each by special muscle fibres, whose function is, in the case of the cardia, to prevent regurgitation of food from the stomach into the œsophagus; and, in the case of the pylorus, to prevent the food from passing into the duodenum before the process of gastric digestion

The Stomach.

1. Œsophagus. 2. Cardia. 3. Greater curvature of stomach. 4. Pancreas. 5. Jejunum. 6. Duodenum. 7. Pylorus. 8. Gall-bladder. 9. Common bile duct. 10. Lesser curvature of stomach.

has been completed. The walls of the stomach consist of three layers. The outermost of these is the peritoneum. The middle layer consists mainly of the muscle tissue, by means of which the stomach is able to empty itself. The innermost layer, which comes into direct contact with the food, is the mucous membrane, and contains the glands which secrete the gastric juice.

### Digestion and Indigestion.

It is necessary to have a clear concept of the functions of the stomach in order to understand the derangements to which it is liable. When food is taken, the gastric juice commences to flow from the glands in the mucous membrane. This juice contains two substances, both of which are essential to proper digestion. These are hydrochloric acid and a ferment called pepsin. By the united action of these the food is softened and partially digested. If either constituent is absent or deficient in amount, gastric digestion will be delayed or imperfectly performed.

In addition to its influence on digestion, the hydrochloric acid acts as an antiseptic, and destroys the majority of germs which are swallowed with each meal.

When gastric digestion is completed, the pylorus relaxes and allows the chyme, as the stomach contents are now called, to pass into the duodenum, where the next stage of digestion commences. But if there are any hard lumps of undigested food in the stomach they cause spasm of the pylorus, and in this way prevent the stomach from emptying itself into the duodenum. This reservation weighs heavily on mankind, and ruins the happiness of many a household; for hard, indigestible lumps in the stomach are by far the commonest cause of dyspepsia in individuals who are otherwise healthy.

It is partly for this reason that man cooks his food. He cannot, with the time at his disposal, chew a raw potato, for example, into such minute fragments that they certainly will not set up excessive pyloric spasm. Solid masses of animal food can be liquefied by the gastric juice, but raw or imperfectly cooked vegetable foods are nearly always a stumbling-block even to the healthy stomach. In addition to the improper selection of articles of diet and their unskilful treatment in the kitchen, another common cause of this species of dyspepsia is insufficient mastication, whether due to habit or to the absence of a complete set of molar teeth.

In the course of fevers or debilitating illnesses the secretion of gastric juice may be much diminished, so that there is a difficulty even in digesting animal foods such as meat and milk, and care must be exercised to give only so much food as the patient can assimilate, and it must be of a kind to require little or no mastication, for a sick man cannot be relied upon to bite up his food properly.

It is unfortunate that milk is popularly looked upon as a liquid food, and is often regarded as the best nutriment for the sick. Raw cow's milk is not a liquid food; it is a solid food, and is quite unsuitable for patients whose powers of digestion are at a low ebb. It would be as sensible to give a slice of raw beef as to give a glass of raw cow's milk to such a patient, because in the stomach the milk is liable to form large, tough curds, which require a long time to digest and soften. Often enough, such hard curds, the size of walnuts, may be found in a patient's stomach, and it is a matter for wonder how nature ever manages to dispose of them at all. If milk is to be used it should be boiled first, as by this means the formation of large curds is avoided.

If the food, either on account of pyloric spasm or other cause, is retained in the stomach beyond the normal time, it undergoes fermentation, as the result of which the stomach becomes inflamed and distended with gas. In these circumstances the patient suffers from discomfort and pain, his tongue becomes furred, and, in fact, he has the well-known symptoms of dyspepsia.

Repeated attacks of the kind now alluded to will lead to chronic inflammation of the stomach, or gastritis, which is accompanied by diminished capacity for digesting food; and the repeated distension of the organ causes it at last to become permanently dilated. So that with the lapse of time the stomach finds difficulty in secreting enough juice to digest even the simplest foods, and it also has constant difficulty in evacuating its contents into the duodenum. When this stage of chronic dyspepsia has been reached, the complaint is probably incurable, although the discomforts which it causes may be relieved by careful dieting and by giving antiseptic medicines which prevent the fermentation and consequent distension of the somach. In severe cases daily lavage of the stomach may be necessary.

### Hypersecretion.

There is another common form of indigestion which is due to excessive secretion of hydrochloric acid in the gastric juice. The sufferer from this is usually a young adult, and he complains of "burning" pain in the epigastrium coming on some hours after a meal, and perhaps relieved by taking more food.

In this form the tongue may be clean, and the general nutrition of the body probably does not suffer a great deal. The pain may be cured by giving a little soda dissolved in water, in order to neutralise the excess of hydrochloric acid. An important point about this hypersecretion is that it is often associated with ulceration of the stomach.

### Other Forms of Dyspepsia.

Other varieties of indigestion are those due to (1) irritating articles of diet, including alcohol, very hot drinks, and stimulating condiments; (2) carcinoma of the stomach; (3) general illnesses—for example, phthisis.

### Aids to Diagnosis.

By no means the least part of a nurse's work is to help the medical man to make a diagnosis. In this a careful and intelligent nurse is of the greatest assistance, and her observations may be of the utmost value. Such a one will not hastily dispose of any vomited matter, lest what may be the most important clue to the case is irretrievably lost. Specimens of vomit should always be preserved for the surgeon to inspect and test.

So, too, should a specimen of urine always be kept for analysis. Patients who are uræmic from advanced disease of the kidneys may have a great deal of pain in the epigastrium, accompanied by severe vomiting, and it is easy to be misled into attributing the symptoms to some gastric affection unless the urine is examined.

### The Nurses' Clinic.

### THE CALCULATION OF THE STRENGTH OF SOLUTIONS.

It is a curious but undeniable fact, that many well-educated and otherwise intelligent nurses, find a great difficulty in making very simple calculations in the mixing of lotions and in the measurement of drugs. A correspondent writes to the Editor of The Hospital to ask, (1) "How much carbolic acid 1 to 20 does one take to make 1 to 40, 1 to 60, and 1 to 80, and how much water does one add? How much perchloride of mercury 1 to 500 does one take to make 1 to 1,000 and 1 to 2,000, and how much water does one add?" Now, the answer to these queries is a very simple matter of arithmetic, and one would be inclined to reply, learn arithmetic and use your reason—which, no doubt, would be good advice, but scarcely practical help. It is no good denying that a great many nurses are, like the correspondent in question, "Puzzled," and have no head for figures, as they say and want "an easy way to calculate quickly the different solutions of lotions." To those nurses this clinic is addressed, in the hope that it may be a help.

First of all let us get a clear understanding of the meaning of the terms used. A 1 *to* 20 solution of carbolic acid, or carbolic acid lotion 1 to 20, means that the lotion is made by adding 1 part of carbolic acid to 20 of water. A 1 *in* 20 solution is made by mixing 1 part of carbolic acid with 19 of water—20 parts in all, 1 part in 20 being carbolic acid.

Now we must dismiss the idea of quantity from our minds. This is not a question of quantity, but of proportion. We are not dealing, for instance, with any particular amount of carbolic lotion when we say that its strength is 1 to 20; we can take a teaspoonful or a gallon from a jar of it, but its strength, that is to say, the proportion of carbolic acid to that of water, will remain the same—1 part carbolic to 20 of water. It is here "Puzzled" goes astray when she asks "how much 1 to 20 does one take to make 1 to 40?" The question is not how much 1 to 20 does one take, but how much water does one add to what one has taken to convert a 1 to 20 solution into 1 to 40, 1 to 60, or 1 to 80. A moment's thought will show, that to make 1 part carbolic to 20 of water into 1 part carbolic to 40 of water, one must double the quantity of water. If one takes a teaspoon of 1 to 20, one must therefore add a teaspoon of water, if one takes a gallon of 1 to 20 one must add a gallon of water to produce 1 to 40 from 1 to 20. Three times as much water as lotion for 1 to 60, four times as much for 1 to 80. So that a 1 to 20 solution is twice as strong as 1 to 40, three times as strong as 1 to 60, and four times as strong as 1 to 80. And it is easy enough to see this if one remembers that the increase in the figures—20, 30, 40, 60, 80—represents that much increase in the amount of water added to the 1 solitary part of carbolic. Now for the reverse process. Supposing you had only a 1 to 40 solution in the ward, and the house surgeon asks you for 1 to 20, or a very strong lotion, 1 to 10. You will now see, that if you were to add 1 part carbolic to 1 to 40, you would make it into 2 to 40, or 1 to 20. If you were to add 3 parts carbolic to 1 to 40 it would become 4 to 40, or 1 to 10. This, of course, is merely by way of example, because pure carbolic acid, of which the lotion is made, would not be available in a ward; the strong solutions would be made in the dispensary.

Now, do we clearly understand this? If a ward of 20 beds had one nurse at night, the proportion would be 1 to 20 beds, if there were two nurses on duty in the afternoon the proportion would be 1 to 10 patients, if four nurses were in the ward in the morning, the proportion would be 1 to 5. The nursing staff of 1 nurse to 5 patients would be four times as strong as 1 nurse to 20.

Having mastered the meaning and relations to each other of a 1 to 20, 1 to 40, 1 to 60, and 1 to 80 solution, let us consider the question of percentage. Five per cent (written

**THE NURSES' CLINIC**—*Continued.*

5%), 10 per cent., etc., are just as frequently used to describe the strength of a solution as are 1 to 20, 1 to 40, and so on. We simply mean by the percentage of a solution, how many parts of the substance—carbolic, perchloride, or whatever it may be—there are to every hundred parts of water or alcohol, or whatever else the solution is made up with. Now, what is the percentage of a 1 to 20 carbolic solution? There is not much difficulty in discovering that. If there is 1 part of carbolic to every 20 of water, it is obvious that there must be 5 parts of carbolic to each 100 of water—5%, 5 per cent. in fact. A 1 to 10 solution, having just twice as many parts of carbolic to the 20 of water, would be a 10 per cent. solution; 1 to 40 would be at the rate of 5 to 200, and so $2\frac{1}{2}$ to 100—$2\frac{1}{2}$ per cent.; 1 to 80, being only half as strong as 1 to 40, must therefore be $1\frac{1}{4}$ per cent.

Now let us go back to our illustration of the 20-bedded wards. Imagine our hospital contains five such wards, 100 patients in all. In the daytime, with 4 nurses to each ward of 20 beds, there would be 20 nurses in the hospital for 100 patients, so that we see that 4 nurses to 20 beds is 1 to 5 or 20 to 100—1 to 5 per cent.

In the afternoon we have half the number of nurses—2 to 20 beds or 1 to 10; there are then 10 nurses to 100 patients, 1 to 10 being therefore 10 per cent.

At night we have only 1 to 20 patients, 5 to the hundred, and so 5 per cent.

So that you will see, that if you increase the number of nurses in proportion to the number of patients, or the parts of carbolic to those of water, you get a stronger nursing staff or a stronger solution—a higher percentage, in fact. If, on the other hand, you increased the number of patients to each nurse, or water to the lotion, you would lower the percentage and weaken the staff or solution. If each nurse had 50 instead of 20 patients there would be 2 to 100—2 per cent. If the 5 night nurses had to manage another hundred patients between them, they would be 5 to 200, or $2\frac{1}{2}$ per cent.

We will now turn to another calculation which sometimes puzzles nurses. What is the percentage of a solution containing gr. j. ad ʒj., or ℥j, or Oj.? One grain to the drachm, ounce, or pint?

First, how many grains are there to a drachm? (They are usually calculated as minims in a fluid form.) These are sixty. One grain to the drachm is written gr. j. ad ʒj. (the particle "ad" meaning "to the," quite a distinct word from "adde," meaning "add there"). One grain to the drachm is 1 grain to 60 grains, 1 to 60, which you will find, if you work it out, to be $1\frac{2}{3}$ per cent., written $1\frac{2}{3}$%. Now let us take one grain to the ounce instead of to the drachm. There are 480 grains to the fluid ounce, so that one grain to the ounce, written gr. j. ad ℥j., is 1 grain to 480, 1 to 480, often calculated in round figures as 1 to 500, which is only one-fifth part per cent.—$\frac{1}{5}$%. A 1 to 500 solution of perchloride of mercury would be approximately gr. j. ad ℥j., one grain to the ounce. A 1 to 1,000 solution, half a grain to the ounce; 1 to 2,000, a quarter of a grain to the ounce.

A drachm to the ounce, ʒj. ad ℥j., is 1 drachm to 8 drachms. Since 8 drachms go to the ounce, the proportion is 1 to 8, 25 to 200, and so $12\frac{1}{2}$%, twelve and a half per cent. A drachm to the pint ʒj. ad Oj. A pint contains 20 ounces, or 160 drachms, so a drachm to the pint is 1 to 160, or $\frac{5}{8}$%, five-eighths of a drachm per cent. An ounce to the pint, ℥j. ad Oj., is 1 ounce to 20, 1 to 20, or 5%, five per cent.

These are a few of the calculations the nurse has most often to make. But there is no easy and quick method of calculating which will obviate the necessity of understanding the meaning of the calculations. If abstract figures are puzzling, the nurse should take concrete things. She might work the sums out for herself, taking peas or shot, and colouring 1 to 5, 10, 20, 30, etc., and count the number of coloured to a hundred plain, or pursue the same plan with drachms, ounces, etc., using one coloured to eight plain for a drachm to the ounce, and so on.

### Incident in a Male Nurse's Life.

MY PATIENT.

I say "my patient," because I think I was the only one who took much interest in him. He was a lad of 19 years, a face strangely feminine, dark, and of foreign nationality, and he was admitted into one of the infirmary wards of a large county asylum, very weak, much emaciated, and with strong suicidal tendencies. After having been put into a nice clean, comfortable bed and examined by the doctor, his name was entered on a piece of parchment, and we nurses in the ward signed the parchment promising never to let him out of our sight until he was passed over to the night staff. My fellow-nurses, after reading the reports which accompanied him and noticing his condition, pronounced him a hopeless case, but I said nothing. In my own mind I had fully determined that I would do my utmost in persuading him to eat, and so get strong, and perhaps well. The doctor ordered various medicines and a slop diet at first, consisting of milk and eggs, beef tea, Plasmon, and Sanatogen, but as he would not open his mouth to be fed with the ordinary feeding-cup, we had for a few days to feed him with the œsophagus tube, and when it was over he would nearly always exclaim in a vexed manner, "What is the use of doing this? I have got to die, so let me alone!" However, I persevered, and the lad got stronger, his bed sores healed, and the doctor thought one morning he might get up a little. It was nothing new for him to get up, for as soon as he was strong enough to stand he would watch us about the ward, and when the opportunity came, it may be when we were engaged in feeding a general paralytic or a cancer of the throat case, he would quietly slip out of bed and rush with his head at the window, smashing the glass, but fortunately never cutting himself severely. When put to bed again, tucked in, and told to be quiet, he would say, "Why don't you let me jump out of the window? I have got to die somehow or other." Well, the doctor's orders were carried out. The patient was dressed and told to sit in an easy wicker chair by the fire, and to be very good. He was no doubt very much better, and I felt quite gratified when he asked to be allowed to help to clear the ward of the flowers and plants for the night. To please him I allowed him to do so, and we had almost finished our task when I heard a terrific smash, and saw him making a resolute attempt to force his head through the window. I felt guilty of having allowed him so much freedom, and also sadly disappointed. He was quickly seized, examined for injuries, reported to the doctor, put to bed, and told to remain there all next day. One day about that time he asked to be allowed to feed himself with a spoon. I consented, and gave him one, but not for long, as he at once tried his best to force the spoon down his throat, so determined was he to destroy himself. Many times I have seen him try to choke himself with his fingers, hold his breath for quite a long time, or try to swallow his tongue, and if he could get hold of a handkerchief he would creep down under the sheets and endeavour to choke himself by tying it tightly around his neck, or by pushing it into his mouth. Again, when he was being bathed, he would try to get his head below water, in the hopes of being drowned, and when he saw his attempts were useless, he would piteously cry, "Do let me die!"

However, one day a great change came over him. He talked to me about his parents, his friends, and his occupation, etc. There was no doubt that he was distinctly improving; his suicidal thoughts seemed to have left him, he was to be trusted with a espoon, and by and by with a knife and fork for his dinner, which pleased him much. He continued to improve, and, after a stay of over six months, he left us for the convalescent ward, where he remained a short time before being discharged, to my great content, as thoroughly cured.

I hope I shall not be considered egotistical if I say that I felt proud of the result of my endeavours, and for the future was inspired with more courage to persevere in my trying work.

### Surgical Aprons.

MOST operating surgeons find that their aprons soon wear into holes at the laundry. I believe this to be due to the use of bleached linen which, owing to the process employed, is never so strong or lasting as the unbleached variety. Also because, as usually made, aprons have too many pleats and folds, making them difficult to wash and iron, and wear badly in consequence. To obviate these defects I have

SURGEON'S OVERALL.

designed an operating apron which is all in one piece and, therefore, can be easily and thoroughly washed, and ironed out quite flat. Procure a length of stout unbleached linen, 48 to 54 inches wide, and for anyone 6 feet high cut off a piece 2 yards long, and mark it out as per the diagram. From one end of this cut down the central crease A for 9 inches; then cut out a circle of cardboard the diameter of the collar usually worn—*e.g.* 5-inch circle for a 16 collar, and use this to mark and cut out a circle D at the end of the 9-inch slit A: 23 inches from the same (or top) end, mark off a line on each side 17 inches inwards (BH and JC) from each selvedge, and cut along these lines to form the sleeves. The linen removed from the circle D makes small triangular gussets to stitch in at M and N. Hem top and bottom ends,

CUTTING-OUT DIAGRAM.

and along each of the slits AD, BH, and CJ. Fix tapes to A to fasten at the back of neck and stitch a collar of cambric or linen 2 inches deep all round the circle D. This latter is to tuck in all round the surgeon's own collar to prevent soiling the latter and to keep the apron from dropping away from the neck. Run tapes through the hems E and F to reef up the sleeve edges above the elbow, and put tapes on at H and J to complete the sleeve fastening. Long tapes attached to K and L are drawn round the waist and fasten in front. For shorter people use linen 48 inches wide and $1\frac{1}{2}$ to $1\frac{3}{4}$ yards long, and so on in proportion. This apron is easily put on, gives absolute freedom of movement, covers the operator very completely, and can be folded in a small compass.

### ARTIFICIAL SPONGES.

I find that squares or oblongs knitted from No. 6 or 8 three-ply unbleached Strutt's cotton on No. 11 needles make most excellent substitutes for sponges. They are soft, very absorbent, and can be boiled—after cleansing from blood in cold water—with impunity. They are cheap, and therefore if thought necessary, may be burnt after use. For a 4-inch square swab I believe 44 stitches are necessary, beginning at one corner, increasing to 44 in the middle and then diminishing to the opposite corner. For use in the abdominal cavity I believe that all artificial sponges should be covered with fine cambric or lawn to minimise damage to the delicate peritoneal coverings and avoid entanglement with the omentum. Two or three thicknesses of Turkey towelling placed inside a cambric bag and then quilted all over or only from corner to corner to prevent shifting is an excellent abdominal sponge cloth. An ordinary fine linen serviette is also useful for packing bowels away from the field of operation and is very smooth of surface.

### Devon and Exeter Hospital, Exeter.

At the request of the Nursing Committee, Mr. Dick, the Secretary of the Royal National Pension Fund for Nurses, explained the aims and objects to a meeting of nurses of this hospital, which has quite recently federated with the Fund. The chair was taken by Mr. S. P. Pope, Chairman of the Nursing Committee, and amongst those present were Mr. P. J. Kendall, Hon. Treasurer; Messrs. H. W. Michelmore, Martin, and Munk; Dr. Stirk, the house surgeon; and the matron, together with about fifty sisters and nurses.

After Mr. Dick's address, Mr. Michelmore told the nurses that the Nursing Committee, appreciating the work of the nursing staff, had considered the best way to help them, and, following the Divine principle, they had decided to assist those who were ready to help themselves. It was the duty of everyone to provide for the rainy day, which in the case of the nurse is longer than that of the average woman, and to render oneself independent of friends or the Poor-law.

The Committee had considered the best channel, and had had the Fund thoroughly examined by a leading actuary, from whom they had received a thoroughly satisfactory report. Mr. Michelmore read a letter from the Secretary of the Bristol Hospital, recommending the Fund, as that hospital was also federated with the Fund. He (Mr. Michelmore) then went on to explain the advantages, pointing out that the cream was not skimmed off, there being no entrance fees, no commission paid to agents, no directors' fees to pay, although the Board was one of the strongest financial combinations in London; there were no shareholders to receive dividends; the working expenses were under 4 per cent., against the average of insurance companies, which was about 15 per cent.; in addition to the profits made by the working of the Fund there was a Donation Bonus Fund, which also helped to increase the pensions which the nurses have paid for; and, beyond all this, a Benevolent Fund, to assist those who, from no fault of their own, found themselves in distress. He explained to the nurses that the money was always their own, and they could draw it out with compound interest at $2\frac{1}{2}$ per cent. per annum; that they would never receive back less than they had paid in, and that, after a certain time, there was no charge at all for working expenses; at the same time it was far better to draw the annuity instead of the principal.

Mr. Michelmore showed his audience a bundle of £5 notes, and explained that the Committee was prepared to give one of these notes each year to pay the premiums for each nurse who contributed as much; then, tearing the note in half, said that if the nurse paid only £2 10s., the hospital would similarly contribute as much; while in the case of sisters, nearly a note and a half (£7) would be paid by the Committee on the sister doing as much herself.

He said that nurses elsewhere were able to save through the Fund, therefore why not those of the Devon and Exeter Hospital, and that the requisite self-denial always tended to develop and improve the character of the individual.

A large number of questions were asked by the nurses present, with the result, doubtless, that many nurses of this hospital will become members of the Fund.

### Wants and Workers.

"A. M. H.," 12 Rose Terrace, Perth, writes that she has a black summer cloak, with cape, length back 58 inches. It is nearly new, but having had to change her uniform, she has no further use for it. She would be glad to give it to any nurse to whom it might be useful, on hearing particulars of position.

### The Government and Registration.

On Thursday afternoon last week the Earl of Crewe, in the capacity of Lord President of the Council, received a deputation on the subject of the registration of nurses.

The deputation was introduced by Mr. H. J. Tennant, M.P., who urged that it was most desirable to carry out the recommendations of the Select Committee on the Registration of Nurses, which he had presided over for two Sessions, as it had been abundantly shown that a large number of persons who undertook the business of nursing were not qualified. It would, he contended, be an advantage if they had voluntary registration, and that those who qualified themselves should be placed upon a list, and it was assumed that preference would be given to them by those who required qualified nurses. There was no desire to prevent others who were less qualified from being employed as nurses by those who wished to have them, although they were not registered or certificated.

Sir James Crichton Browne, representing the Royal British Nurses' Association, said that the Association had for some time felt the need for legislation, and it had, in fact, anticipated it, to some extent, by keeping a list of fully trained and competent nurses. There had, it was true, taken place an enormous improvement in the profession of nursing of recent years, and that without State aid. But the fact that such an improvement had taken place without State aid was just the reason why State aid was now necessary. Nursing had become a fine art, calling for special skill, technical ability, and cultured insight. But the public were not a judge of these qualifications, and there were undoubtedly a large number of spurious nurses and moral delinquents going about, against whom it was necessary to afford protection. Registration, therefore, would prove advantageous, not only to the public, but also to the genuine nurses themselves.

Mr. Hobhouse said he hoped that legislation would not interfere with the work of cottage nurses, many of whom were of great help in rural districts.

Lord Crewe, in reply, said that the Government had a full programme of legislation, and he could not hold out any hope that they would take the matter up during the present Session. But he fully recognised the importance of the subject, and if a private member brought forward a Bill in either House of Parliament, the Government would give it benevolent attention.

### East London Nursing Society.

The annual meeting of this Society was held at the Mansion House last Tuesday. There was a good attendance, but various disappointments awaited the meeting. The Lord Mayor was unable to take the chair, as he had been called away to an important conference. Sir Walter Phillimore was also prevented from coming, and Sir Edmund Hay Currie was ill and could not be there.

The chair was taken by Alderman Sheriff Smallman, who, in a few introductory remarks, said that the Society had been formed in 1868, and had done an enormous amount of good in the East End of London, where nursing was so much wanted. In looking through the report he noted that the 30 nurses employed by the Society had been most assiduous in the discharge of their duties, and had paid about 4,000 visits each, which meant that they had attended on more than 100,000 occasions those who needed help. The

Royal Amateur Society had last week held a sale of works of art, with the very satisfactory result that a handsome donation would be given to this Society. There was nothing, in his opinion, that appealed so much to Britishers as nursing, because all sooner or later felt the benefits of it. The Society was very desirous of having more than 30 nurses, and he hoped that the result of this meeting would be that they would be able to add to the number. The income last year was about £2,500. This sum, divided by the number of patients, showed that on an average it cost only ten shillings to relieve the wants of one patient. He hoped the subscriptions would increase so as to enable the Society to deal with a larger number of patients.

Dr. Bertrand Dawson, physician to the London Hospital, moved the adoption of the report, and remarked that there were very few callings in which so much was expected as in that of a nurse. She had to be physically strong, intelligent, quick, ready, prompt, sympathetic, unselfish, and devoted. Those who possessed all these qualities were a very great influence for good. The nurses of this Society had to do their work under very adverse conditions. The presence of a nurse did not mean only the alleviation of pain, but often saved the lives of the sick poor. From a merely economic standpoint nothing could be of greater social benefit than the supply of nurses. The nurses not only cured sickness at the time, but prevented sickness in the future. They taught people new ideas and enlarged their minds, and proved to them that because people were ill they did not cease to need air and soap and water. They might be called the pioneers in preventive medicine, as well as educationalists.

The resolution was seconded by the Rev. Prebendary Dalton, who gave instances of the astonishing ignorance of the poor and of the great benefits conferred by the nurses.

Sir Thomas Hewitt, K.C., proposed the re-election of the vice-presidents, councils, and honorary officers. He knew something about institutions of this kind, as they had started a similar one in his own county of Devon, which had proved an immense success. There were at least 100,000 poverty-stricken people in the East End of London who, when they were ill, needed the help of those who were better off. Being a lawyer, he looked at the matter from a commonplace point of view, and the fact of the meeting being in the City had made him reflect that in old days the merchants and workers in the City lived there and contributed towards the support of their poorer neighbours. All this was changed now, and the dwelling-houses were taken by companies who did not dispense their money with the same freedom as the old inhabitants. Being at the head of cne of the largest accident insurance companies, he had suggested at their board meeting that day that they should make a contribution to the East London Nursing Society, and they had agreed to do so. This example might be followed by other City companies. The non-sectarian character of the Society was, to his mind, its greatest merit. It required and deserved a much greater amount of support.

The resolution was seconded and carried.

Sir William Quayle Jones, Chairman of the Committee of Management, who followed, said that this Society did a very great work in bringing into touch classes that would not otherwise come in contact with each other. Their assistant ladies went round with the nurses and rendered a large amount of personal service, supplying those comforts so sorely needed in times of sickness. They had lost in the last ten years over £300 in annual subscriptions. Unless they obtained greater support this year they would be obliged to reduce the number of their nurses.

A vote of thanks to the Chairman brought the meeting to a close.

### The Nurses' Bookshelf.

**Lectures on the Nursing of Infectious Diseases.** By Dr. F. J. Woollacott. (Scientific Press. 2s. 6d. net.)

Three useful chapters on infection, the prevention of infectious diseases, and their general management form a prelude to an excellent handbook for the fever nurse. The striking feature about it is its great practicality; the directions given are plain and straightforward, and, with the hints scattered through the book, are sufficient to put an already trained nurse in the way to manage a case which may be unfamiliar to her. The chapters on diphtheria describe its early symptoms, its treatment, and the chief dangers arising from it, with such complications as infection of the conjunctiva, etc. Next comes a description of the antitoxin treatment, including the rashes that may result therefrom; another chapter is devoted to diphtheritic croup, with its operations; and finally a chapter on diphtheritic paralysis. The nurse is given full directions as to feeding and the general treatment of the case, and many useful "tips" in addition. For example: "The child should be talked to and encouraged to play with his toys, and it is a good plan (in cases of tracheotomy) to get him to blow about a feather, so that breathing by the mouth may be restored as soon as possible." And, again, in paralysis the patient "should be kept absolutely at rest, and all mental excitement must be prevented, as any emotion, pleasurable or otherwise, might be followed by fatal collapse." The chapters on enteric nursing are equally good. The author impresses the need of absolute rest and plenty of sleep. He recommends that the patient should not be waked for food, except by the doctor's direct orders; and advises a diet of two or three pints of milk, diluted and flavoured, if liked, and two or three pints of plain or toast water or lemonade. He adds: "In general it is advisable to introduce as much variety as possible, for a pure milk diet soon becomes monotonous and distasteful. Of recent years there has been a tendency to make still further modifications, and many medical men now allow solid food in the less serious cases when they consider the condition of the patient suitable." Scarlet fever, whooping cough, and measles are also dealt with in the course of the book.

**Massage and the Original Swedish Movements.** By K. W. Ostrom, from the University of Upsala. (Sixth Edition. H. K. Lewis. 3s. 6d. net.)

A course of lectures, originally given before the nurses of various American training schools, has been reproduced in this book, with more than a hundred illustrations, showing, as well as can be done in pictures, the various movements used in massage and the so-called Swedish movements. The book is not intended for nurses who are studying the subject for the first time; it is not a text-book of bones and muscles; it is rather for those who, having gained their certificate, wish to make use of their knowledge. The author expressly deprecates the idea that massage treatment can be self-acquired. He says in his introduction that "manual treatment for disease has to a certain extent existed since the creation. Man had, by instinct, acquired the art of manipulation long before Nature yielded her secrets in medicine. In Sweden, even at the present time, certain manipulations are used among the peasants for cramps, swellings, etc. The Swedes seem never to have lost the art but recently revived in other countries." Massage was commonly practised, too, by the Chinese 3,000 years ago, by the priests of ancient Egypt, and by the Hindoos. But it was only in the eighteenth century that Dr. Ling, a Swede, raised the treatment into a scientific remedy. Directions are to be found for massage of every important organ in the body—for the face, head, and even for the eye. With regard to this last there is a not useless caution that it should be given only by order of a physician. There are excellent instructions for self-applied movements, as for increasing the capacity of the chest and for the cure of constipation.

Notes on Surgery for Nurses. By Joseph Bell, M.D., F.R.C.S.Edin. (Oliver and Boyd.)

This useful little volume, which by permission is dedicated to "Florence Nightingale, Chief of the Nursing Staff," has now reached its sixth edition. It will be particularly welcome to all Edinburgh nurses, where the name of Dr. Joseph Bell is a household word, and the work has been rendered more valuable because it has been thoroughly revised, and by the addition of an appendix treating of the important questions raised as to the Relation of the Trained Nurse to the Profession and the Public.

The Bracebridges. By Sarah Tytler. (John Long. 6s.)

The love affairs of the Bracebridge girls, whose father, having failed in the hereditary occupation of stockbroking, looks to his children to keep the home going, is the subject of Miss Tytler's new novel. In spite of the limits to their aspirations imposed by a suburban circle and the possession of parents described as "a thoroughly drab-coloured couple of a subdued type," the girls, with their intelligence and good looks, make their way, and rise above the obstacles which beset their progress. "The Bracebridges" adds another volume to the library of domestic fiction to which Miss Tytler is an old and valued contributor.

### Everybody's Opinion.

[Correspondence on all subjects is invited, but we cannot in any way be responsible for the opinions expressed by our correspondents. No communication can be entertained if the name and address of the correspondent are not given as a guarantee of good faith, but not necessarily for publication. All correspondents should write on one side of the paper only.]

### NIGHT NURSES OF WHITECHAPEL INFIRMARY.

"A Whitechapel Nurse" writes: I thank you for the insertion of my letter of last week, but regret that my letter was not clear to you, or misread. The grievances referred to my experiences while at Constance Road Workhouse, not Whitechapel. I shall be very glad if you will please correct this in your next issue.

"A Whitechapel Sister" writes from the Whitechapel Infirmary: With reference to the letter published in your last week's issue purporting to come from a Whitechapel nurse, may I be allowed to state that it is a gross untruth from beginning to end. The person who presumes to write such a letter has never been on the staff of this infirmary, or I am sure nothing would have induced her to write it. In the first place, the time stated is quite incorrect. The nurses have four months' night duty, with one night off duty every month, with afternoon passes during the week and on Sundays. The food allowed is most excellent and well served, and never lacking in quantity. Conversation, when off duty, is never restricted. Many of the nurses, and especially night nurses, subscribing to a daily paper, as well as many of the leading magazines, conversation is never lacking, and is often most interesting and amusing. I am sure you could not find a happier set of night nurses in any other hospital or infirmary training school in London. Those in authority are always most kind and considerate, and do their utmost to promote the welfare of the nurses. Many of us consider it a great privilege that we have been allowed to work in Whitechapel among the very poor and needy, and hope that our influence and work will still live on for good to those around us long after we have left Whitechapel. If every nurse had this aim in view—namely, to do her work with all her might and strive to spread a good influence around her wherever she goes, always remembering to look on the bright side of things, knowing that her colleagues are human as well as herself, each striving to do all things well—then indeed we should have no untruthful grumblers amongst us.

"A Whitechapel Infirmary Night Nurse" writes: With reference to the letter which appeared in your issue last week from a supposed Whitechapel nurse: As a night nurse myself in the Whitechapel Infirmary, I beg to state that her letter is entirely untrue, both regarding the off-duty time and the food, which is excellent in quality and quantity. Our conversation is never restricted at meal times. Personally speaking, I thoroughly enjoy those times with my fellow-nurses, and I am sure those in authority do their utmost to prompt our happiness and comfort.

"E. G. T." writes: Will you allow me to say that a person writing to you last week calling herself "Whitechapel Night Nurse" was never in this institution; every statement she makes is untrue and inaccurate. Everything possible is done for the nurses' welfare and comfort. Naturally the whole staff is most indignant that such untruths should be written of one of the best infirmary training schools.

### DR. RHODES AND THE CENTRAL MIDWIVES BOARD.

"A Chorlton Union Hospital Nurse" writes: I read your paper every week with interest. Last week in your notes on news from the nursing world you mention that Dr. Rhodes makes an attack on the Central Midwives Board. Dr. Rhodes has every reason to say that the Board have treated Poor-law institutions with contempt, he being one of the Guardians of the Chorlton Union Hospital. The Board have at last recognised the infirmary as a training school for midwifery, but they were a long time in doing so, and the Guardians went to a great amount of trouble and expense to get the midwifery wards recognised. Previous good results did not seem to count at all with the Central Midwives Board. We average 150 cases a year, and for the last five years we have not had a case of ophthalmia or septicæmia; in fact, results in all ways have been proved to be good. The pupils trained for the London Obstetrical Society were most successful, 24 out of 26 having gained the certificate in the last 18 months of the Society's existence. The first five pupils from here went in for the recent examination of the Central Midwives Board in Manchester, and four of the five passed, which proves that the Board did right to recognise the teaching of midwifery here.

### OVERWORKED PROBATIONERS.

"A Provincial Nurse" writes: In reply to "X. Y. Z.'s" criticism *re* overworked probationers, am I justified in again writing? Opinions were invited from all quarters, and I plainly stated "my own experience" as a provincial nurse, and as such tendered it, not wishing to give the impression that I thought it the same all through the country. Each must admit a nurse careless as regards personal cleanliness deserves to suffer for her negligence; also, persons with nerve and of neurotic temperaments ought never to enter a hospital ward, and the sooner they are weeded out the better. As regards the sad case again quoted, I certainly fail to see where the novelty of the case lies. In all probability she may have been one of a body of nurses who would never speak, "much less complain," of anything of the kind, unless questioned. I have never had the misfortune to be off duty five minutes (beyond my off duty) since entering hospital; but I have heard many nurses who have had serious illnesses say that they would rather die at their work than fall sick and have to be nursed in the same hospital. Pretty sitting-rooms, pianos, etc., have not fallen to my share during my short career. To those nurses who have these comforts and recreations provided, and who fail to appreciate them, I say they do not know how to count their blessings. Last, but not least, comes the sigh for "the good old days of 20 years ago." Ah, well, probably 20 years hence I shall heave a big sigh for the same reason; but I trust with 20 years' experience that the country then may be able to number as many efficient nurses as it can now.

"Unprejudiced" writes: Will you kindly allow a space in your interesting paper for one who is neither probationer nor matron, though hoping in time to become the latter? As "X. Y. Z." remarks, there are two sides to every question, and even the overworked "pro." will admit it. "X. Y. Z." further remarks "Hospital finances will not permit a superfluity of nurses to relieve those who have a cold, a bad finger, or a headache." No; that is not expected, even by the unreasonable "pro." Nurses are not kept "in stock," if I may use the expression, for those who are below par; but it might be possible that the nurses working in the same ward with the nurse who has "a cold" are willing to do her share of the work if "Matron would only allow the nurse to go off duty." A cold cannot be called a serious illness; yet it makes one unfit for the duties of a nurse, or indeed any duties. There are different forms of a cold, as there are different stages of any other disease, and the fact that one person can go about with a cold is not a reason that everyone should or could do the same. My own experience of nurses is that they do not report themselves ill until absolutely obliged. I have known nurses to be in an advanced stage of enteric before the fact was found out, because they tried to screen the fact that they were ill. Care should be taken to avoid driving nurses to this extreme. According to "X. Y. Z." the nurse who suffers from headache should be dismissed as unfit for hospital. Are we to conclude that a headache is incurable, or else an affliction foreign to nurses? People who suffer from "nerves" should not take up nursing, says "X. Y. Z." What of the nurse who begins nursing in vigorous health, but finds, after two or three years, "she is not what she used to be," that she is not able to stand the strain or the difficulties as patiently as she used to. I think that the strongest of us have felt this at times; yet are not really ill. What would "X. Y. Z." do in such a case? Dismiss the nurse, I presume, when perhaps a day or two of rest would make her all right. I fear that if "X. Y. Z." had the choosing of nurses our large ranks would be very much thinned, and the suitable applicants few indeed. It is to be hoped that the majority of nurses in the present day are not so "fragile, neurotic, and self-pitying" as "X. Y. Z." would imply. Matrons of the present day take a keener interest than ever in their nurses. As "X. Y. Z." adds, in nearly all hospitals the nurses are provided with pretty sitting-rooms, tennis courts, and various other things, and they take it for granted that it should be so. And why not? Does not the matron enjoy all these privileges in a greater degree, as her position entitles her to? Does she not take it for granted that it should be so?

### TRAINED NURSES AND UNTRAINED WOMEN.

"A St. Thomas's Nurse" writes: May I say a few lines on the same old subject of untrained women? I consider medical men to blame a great deal for this reason. Do they inquire where a nurse has received her training, or whether she has been trained at all? No. Why not? I consider it great thoughtlessness on their part. As long as a woman wears uniform of some shape or colour she is considered a trained nurse. I know a woman now, once a nurserymaid and general servant, having £40 per annum to look after and keep clean an old lady, and I hear that she thinks of commencing "daily visiting nursing." It is true that she was for some time assistant at a convalescent home—but is that being trained? Why should our bread be taken from us after years of toil as probationer, sister, or matron? I am a St. Thomas's Hospital nurse, and can say mine was hard work from the day I entered. I am also a certificated masseuse, with three months' training. But I know a girl who went to learn massage one fortnight, and some kind of a certificate was given her, and she goes out as a certificated masseuse. I do not say that the untrained woman cannot nurse. That is not the point. Let all nurses who can give up outdoor uniform. I consider that it is a disgrace. Dozens of women pass my dwelling daily dressed in all kinds of coloured uniform. Nothing but nursery-maids pushing mail-carts and going errands. I heard a surgeon say quite recently, "It was bad form to don outdoor uniform." What should we say if the clergy and doctors could not state the school or college they came from, and if the former wore their dress without their University training? Cannot our kind editor do something for us? Daily I hear terrible tales, and they are only too true. I know of a fever hospital not far off with these untrained women, and pretty pranks they play. They are a disgrace indeed to womanhood.

### Appointments.

[No charge is made for announcements under this head, and we are always glad to receive and publish appointments. The information, to insure accuracy, should be sent from the nurses themselves, and we cannot undertake to correct official announcements which may happen to be inaccurate. It is essential that in all cases the school of training should be given.]

Belvedere Hospital, Glasgow.—Miss Sarah G. Aitken has been appointed matron. She was trained at the Western Infirmary, Glasgow, and has since been night superintendent at the General Hospital, Wolverhampton, and matron of Bradford Incorporated Nurses' Institution.

Chelsea Hospital for Women, London, S.W.—Miss Alice Clarke has been appointed assistant matron, Miss Edith A. Speight night sister, and Miss Eeva Neate charge nurse. Miss Clark was trained at the Hospital of St. Cross, Rugby, and at the General Infirmary, Leeds. She has since been sister of the theatre and male ward at the Victoria Hospital, Keighley. Miss Speight and Miss Neate were both trained at the Victoria Hospital, Keighley, where they have since been staff nurse and night charge nurse respectively.

Combination Hospital, Johnstone, N.B.—Miss Mabel Spencer has been appointed sister of Stuart Fever Pavilion. She was trained at St. Bartholomew's Hospital, London, and has since been nurse and sister at the City Hospital, Sheffield.

King Edward VII. Sanatorium, Midhurst.—Miss Blanche Trew, R.R.C., has been appointed matron. She was trained at University College Hospital, and has since been charge nurse at the National Hospital, Queen Square, Bloomsbury; sister at Derby General Hospital; assistant superintendent of the Nurses' Co-operation; and matron of Truro Infirmary. She also served in South Africa for 18 months as a member of the Army Nursing Service Reserve.

Plaistow Fever Hospital.—Miss Florence M. Day has been appointed staff nurse. She was trained at St. Mary's (Islington) Infirmary, has been engaged in fever nursing at the Cambridge Sanatorium, and has done private nursing in Leeds.

Royal Cornwall Infirmary, Truro.—Miss E. F. Scott has been appointed matron. She was trained at Bolton Infirmary, and has since been night superintendent in the same institution; she has also been matron at Bury Dispensary and Hospital.

Royal Infirmary, Derby.—Miss C. Alcock has been appointed out-patient sister. She was trained at Coventry and Warwickshire Hospital, Coventry, where she has since been sister in medical ward and sister in out-patient ward.

Royal Infirmary, Preston.—Miss Flora Jones has been appointed night sister. She was trained at the Royal Infirmary, Preston, and has since been night sister at Princess Alice Hospital, Eastbourne, and charge nurse of the theatre at the Royal Infirmary, Preston.

St. Mary (Islington) Infirmary.—Miss Edith Smith has been appointed sister. She was trained at Islington Infirmary, where she has since been staff nurse.

Southampton Branch of Queen Victoria Jubilee Institute.—Miss Emma Dudley has been appointed superintendent. She has been a Queen's Nurse since July 1897, and recently she resigned from the superintendentship of the Gloucester Home.

Thanet Isolation Hospital, Ramsgate.—Miss Rosamund Pearson has been appointed charge nurse. She was trained at the Royal Free Hospital, London, and has since done private nursing and been a charge nurse at the Isolation Hospital, Biggleswade.

The Royal National Orthopædic Hospital, London.—Miss Mary E. Pinsent has been appointed matron. She was trained at the London Hospital, and has since been sister and matron of the late Royal Orthopædic Hospital. She was appointed assistant matron at the late National Orthopædic Hospital during the progress of amalgamation, and in consequence of Miss Hole's enforced absence has for the past year been acting as matron of the amalgamated institution.

### Presentations.

Crumpsall Infirmary, Manchester.—Last Friday a presentation was made to Miss Bertha Dutton by the medical and nursing staff of Crumpsall Infirmary on her retirement from nursing work. Sister Dutton has been in charge of the maternity ward for the last 27 years, and a large number of nurses have been trained by her in midwifery and monthly nursing. Past and present nurses joined in the gift, showing the esteem and affection in which Sister Dutton was held. A purse containing 15 sovereigns was presented by the matron, Miss Girdlestone, and all joined in wishing her many years of happiness after her long and useful career as a nurse.

Royal City of Dublin Hospital.—Miss T. J. Tanner has been presented, upon her departure to Bournemouth, with a solid silver toilet set by the nursing staff of the Royal City of Dublin Hospital, including several old sisters and nurses of the institution.

### Novelties for Nurses.

(By Our Shopping Correspondent.)

### STEPHENSON'S FURNITURE CREAM AND FLOOR POLISH.

(Stephenson Brothers, Limited, Bradford, Yorks.)

It seems hardly worth while nowadays to follow in the footsteps of the old-fashioned housewife and concoct our own furniture cream, since Messrs. Stephenson have invented such a satisfactory and inexpensive one. The great secret of success is to use very little, and to finally rub the polished wood or japanned ware with a piece of dry flannel. The best possible result will thus be obtained with the minimum expenditure of time and energy. The floor polish produced in red-topped tins by the same manufacturers is proving an equal success with the domestic staff, and is now being used in some of our largest public institutions. It quite supersedes the time-honoured wax-and-turpentine-in-a-jam-pot system, giving much more quickly an equally durable polish to stained boards.

### Notes and Queries.

**REGULATIONS.**

**The Editor is always willing to answer in this column, without any fee, all reasonable questions, as soon as possible.**

**But the following rules must be carefully observed.**

1. **Every communication must be accompanied by the name and address of the writer.**
2. **The question must always bear upon nursing, directly or indirectly.**

**If an answer is required by letter a fee of half-a-crown must be enclosed with the note containing the inquiry.**

*Rashes in Diphtheria.*

(183) I have a diphtheria patient who is now in the fourth week of disease. On three occasions he has developed a rash after a simple enema. Of course, I reported the rash, and the Medical Officer wished him isolated for scarlet. However, he has since decided it is septic. I mentioned before my nurses and a fellow-matron that I had seen a similar rash after an enema several times during my training. None of them seemed to think it possible. I should be so glad of your valued advice as to whether I am mistaken.—*Moonraker.*

A rash is a fairly frequent consequence of an enema, and is of no especial significance. Also an urticarial rash often follows the injection of antitoxin. In the case you mention, without knowing more details, it is not possible to decide if the rash was due to the enema or to the antitoxin.

*Training.*

(184) A young lady, aged 20-21, is anxious to adopt nursing as a profession. She has had no training yet, except in housework and management. Will you tell me what course she had better follow?—*Medical Man.*

She cannot enter at a good hospital for general training till she is 23. But if she prefer, she can gain experience in a children's hospital earlier, though many of the best training schools only receive probationers without previous training. For further particulars consult "How to Become a Nurse," to be obtained at the Scientific Press, 28 Southampton Street, Strand, London, W.C.

*Country District.*

(185) I am a nurse with two years' general training and certificate for massage, and am anxious to do district work in a country parish. Is it essential to qualify for midwifery, or could I get a post without it?—*Ronny.*

It is generally desirable to be qualified in midwifery for country districts, but there are such posts as you desire in districts where two or more nurses are working. You had better advertise.

*Engagement.*

(186) I should be glad if you will give me some information on the following subject. At present I am at a case of sick nursing, and in all probability the patient will require a nurse for some long time. In May I have a maternity case. If the confinement came on before the full time, what would be the right thing to do? Does a maternity case always stand before any other, even if it comes on before the time? Could the maternity patient expect me to leave this case and go to her? Could I ask this patient to release me, or ought I to stay until she can get someone to take my place?—*Rebecca.*

Your maternity patient could not claim you before the date on which you arranged to go, unless the contingency being anticipated you promised to hold yourself in readiness. The claim your present patient has upon you depends on the terms on which you were employed; if for an indefinite period you should certainly remain with your patient until she has found a substitute; but anyhow it would be wise to arrange for leaving a few days before you are actually due at your maternity patient.

*Training.*

(187) I have four years' experience of mental nursing, and hold the Medico-psychological Association certificate; I wish to get a further general training in an infirmary or hospital where a certificate is granted at the end of two years. Where can I go? There seems to be no hospital with two years' training in Scotland.—*C. M. B.*

Nearly all recognised training-schools now require three years' service before giving a certificate, but many hospitals would receive you as a paying probationer, and you would be entitled to a formal testimonial to the effect that you had received two years' training, but it will not rank as a three years' certificate. Consult "How to Become a Nurse."

*Uniform in Theatres.*

(188) Will you kindly advise me on the following: (1) I am nursing a lady who has asked me to accompany her to the theatre when she is convalescent. We shall book for the dress circle. Could I go in uniform? Should I keep my bonnet on? (2) If asked to spend an evening with friends, should I go in uniform? Would grey alpaca dress without an apron be suitable?—*A. P.*

(1) If not convenient to you to wear private dress, you could go to the theatre in uniform; but if in the dress circle you might be asked to remove your bonnet. Of course the desires of your patient in this matter would influence you. (2) You can, if you wish, also wear uniform when spending evenings with your friends.

*Elementary Nursing.*

(189) Can you tell me if there is anywhere in or near Edinburgh where I could get a course of lessons in nursing? I wish to gain experience in washing, dressing, and management of invalids.—*M. M.*

Some cottage hospitals and convalescent homes will give a certain amount of preliminary training to candidates. You had better advertise, or perhaps you would like to attend St. John's Ambulance Association lectures? If so, write to the Secretary, St. John's Gate, Clerkenwell, London, E.C.

*Civil Hospitals, Shanghai.*

(190) Will you kindly tell me to whom I should apply for particulars in regard to nursing in the civil hospitals at Shanghai and Hong Kong?—*H. H. H.*

Write to the Matrons of the Alice Memorial Hospital, of the Government Civil Hospital, of the Victoria Hospital for Women and Children, Hong Kong, or the General Hospital, and Margaret Williamson Hospital, Shanghai.

*School of Tropical Diseases.*

(191) I am taking up Colonial nursing, and expect to go out to the Colonies next year. Could you tell me whether the School of Tropical Diseases, Liverpool, will take me in for three months' training? I could not afford to pay, but would be willing to go to them for six months.—*M. S. P.*

Write and ask the Secretary, and you may gain some valuable information on the subject by writing to the Matron of the Seamen's Hospital, Greenwich, S.E.

*Prison Work.*

Can you kindly inform me if there is an opening for work for trained nurses in any of the large prisons?—*Pathos.*

Write to the Prison Commissioners, Home Office, Whitehall, S.W. We believe that these vacancies occur very rarely.

*Colonial Nursing Service.*

(192) What are the necessary qualifications for a nurse wishing to enter the Colonial nursing service? Three months' training in some special hospital is given, I understand. Is this after a nurse is accepted for the service? Must a nurse, after being accepted, wait idle until there is a vacancy, or is she sent straight away to the Colnies? What is the scale of pay?—*Nurse Isabel.*

Candidates must have had three years' general training, and hold certificates in monthly nursing and midwifery. Write for details to the Hon. Secretary, Colonial Nursing Association, Imperial Institute, S.W.

*Nursing Infants.*

(193) Will you kindly tell me of some institution in London where a nurse can receive a few months' training to enable her to have the care of infants from the month?—*A. M.*

Write for particulars to the Secretary, Dr. Barnardo's Homes, Stepney Causeway; the Norland Institute, 10 Pembridge Square, London; or the Training Home, Flora Villa, The Wrythe, Carshalton.

*Nursing in Canada.*

(194) Can you give me an idea what the nursing outlook is like in Canada (Winnipeg), and if a private nurse would be likely to get on? I should be glad if you could give me some reliable address where I could get information.—*Constant Reader.*

We do not think that your chances would be good. In Canada there are plenty of excellent and fully-trained nurses, and invalids naturally prefer to employ their own countrywomen. You might write to the Victorian Order of Nurses for Canada, 578 Somerset Street, Ottawa.

**Handbooks for Nurses.**

| | Post Free. |
|---|---|
| "A Handbook for Nurses." (Dr. J. K. Watson) ... | 5s. 4d. |
| "Nurses' Pronouncing Dictionary of Medical Terms" | 2s. 0d. |
| "Art of Massage." (Creighton Hale.) ... ... ... | 6s. 0d. |
| "Surgical Bandaging and Dressings." (Johnson Smith.) ... ... ... ... ... ... ... | 2s. 0d. |
| "Hints on Tropical Fevers." (Sister Pollard.) ... | 1s. 8d. |

Of all booksellers or of The Scientific Press, Limited, 28 & 29 Southampton Street, Strand, London, W.C.

### For Reading to the Sick.

### REST FOR THE WEARY.

Only the lone surge at my feet
Uttered a soothing murmur sweet,
As every broken weary wave
Sank gently to a quiet grave,
Dying on the bosom of the sea;
And death grew beautiful to me,
Until it seemed a mother mild,
And I like some too happy child—
A happy child, that tired with play
Runs to his mother's arms to weep
His little weariness to sleep.

*Archbishop Trench.*

Of charity saints grew. They were once weak, faulty, sinful; they had their burdens and hindrances, their slumberings and weariness, their failures and falls like us. But now they have overcome. Before long we too may be as they. The longest life, how short! The fairest earthly bliss, how poor! A few short years and all will be over. Then there shall be no more sin and jar, no more infirmities and imperfection. Then we shall have the power to taste of bliss, and the former things will have passed away.

So we come to the end of all our thoughts and toils. For what else were we born, and for what end came we into the world, but to behold the face of God? This is the end for which we were created; to this, as to its source and rest, our being tends. To love God and to die, this is the end of man; or read it in the light of heaven, to love God, and to dwell in God for ever, this is our being and our bliss.

*H. E. Manning.*

The rapidity with which time seems to pass in sleep may serve to indicate the shortness of that time which will exist before the reunion of our soul and body. For our Lord, in speaking of that reunion, seems to hesitate whether to speak of it as a future thing or as one already present and at hand. When He says "The hour cometh," He pauses, as it were, to alter the expresion, and adds, "and now is," when the dead shall hear the voice of the Son of God. For a thousand years are to Him but one day.—*Isaac Williams.*

Above all things, in all things, look unto Jesus the Author and Finisher of thy faith. Do all things through His grace, for Him, looking to Him, as thy everlasting great reward. Let nothing keep thee back from Him. If thou failest, look to Him to uphold thee; if thou stumblest, hold His hand to keep thee; if thou fallest, lie not hopelessly there, but look to Him to raise thee; if by His grace thou doest well, look to Him in thanksgiving that He has helped thee, and pray that thou mayest do better.—*E. B. P.*

Faith, Hope, and Love here weave one chain
But Love alone shall then remain
When this short day is gone;
O Love, O Truth, O endless Light,
When shall we see thy evening bright,
With all our labours done?

*Latin Hymn.*

## Figures and Tables

**Figure f1:**
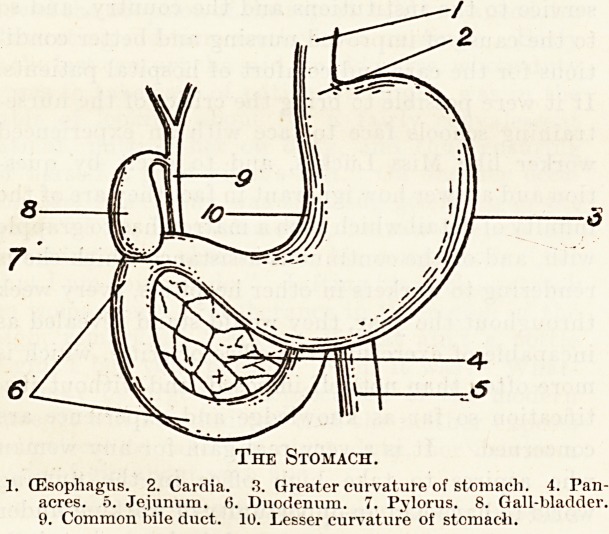


**Figure f2:**
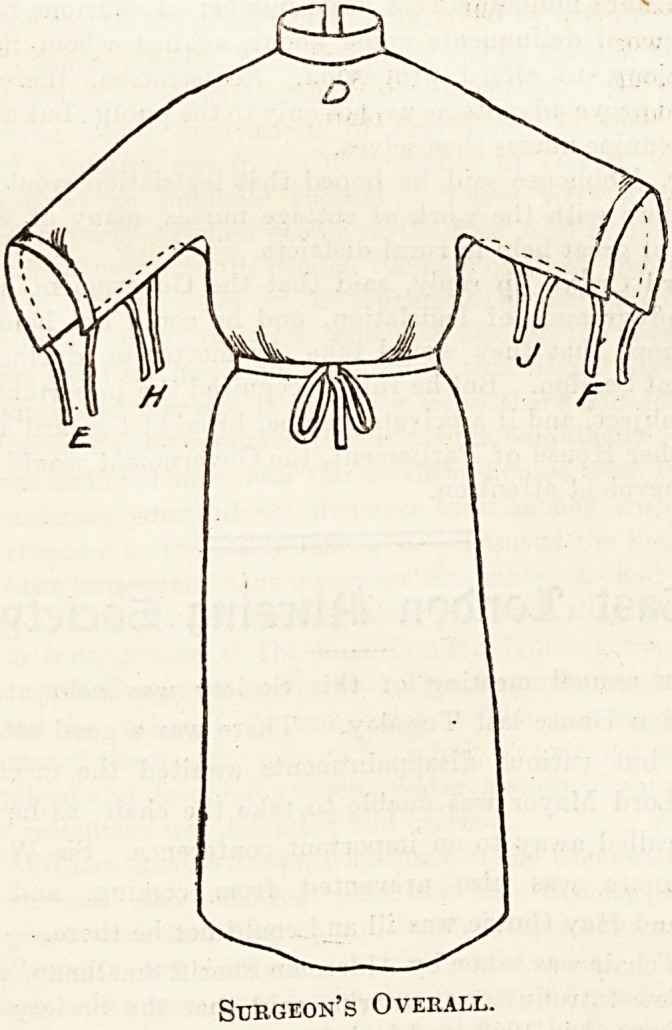


**Figure f3:**